# Supplementary material for: Chromatin profiling identifies putative dual roles for H3K27me3 in regulating transposons and cell type-specific genes in choanoflagellates
Source: bioRxiv. 2024 May 30:2024.05.28.596151. Preprint. [Version 1] doi: 10.1101/2024.05.28.596151 (PMC11160669; doi:10.1101/2024.05.28.596151)
Supplement: Supplement 2 [file media-2.pdf]

Supplementary material

Chromatin profiling identifies putative dual roles for H3K27me3 in regulating transposons and cell type-specific genes in choanoflagellates.

James M. Gahan[1,2,3*], Lily W. Helfrich[4], Laura A. Wetzel[4], Natarajan V. Bhanu[5], Zuo-Fei Yuan[6], Benjamin A. Garcia[5], Rob Klose[2], David S. Booth[1,7,]

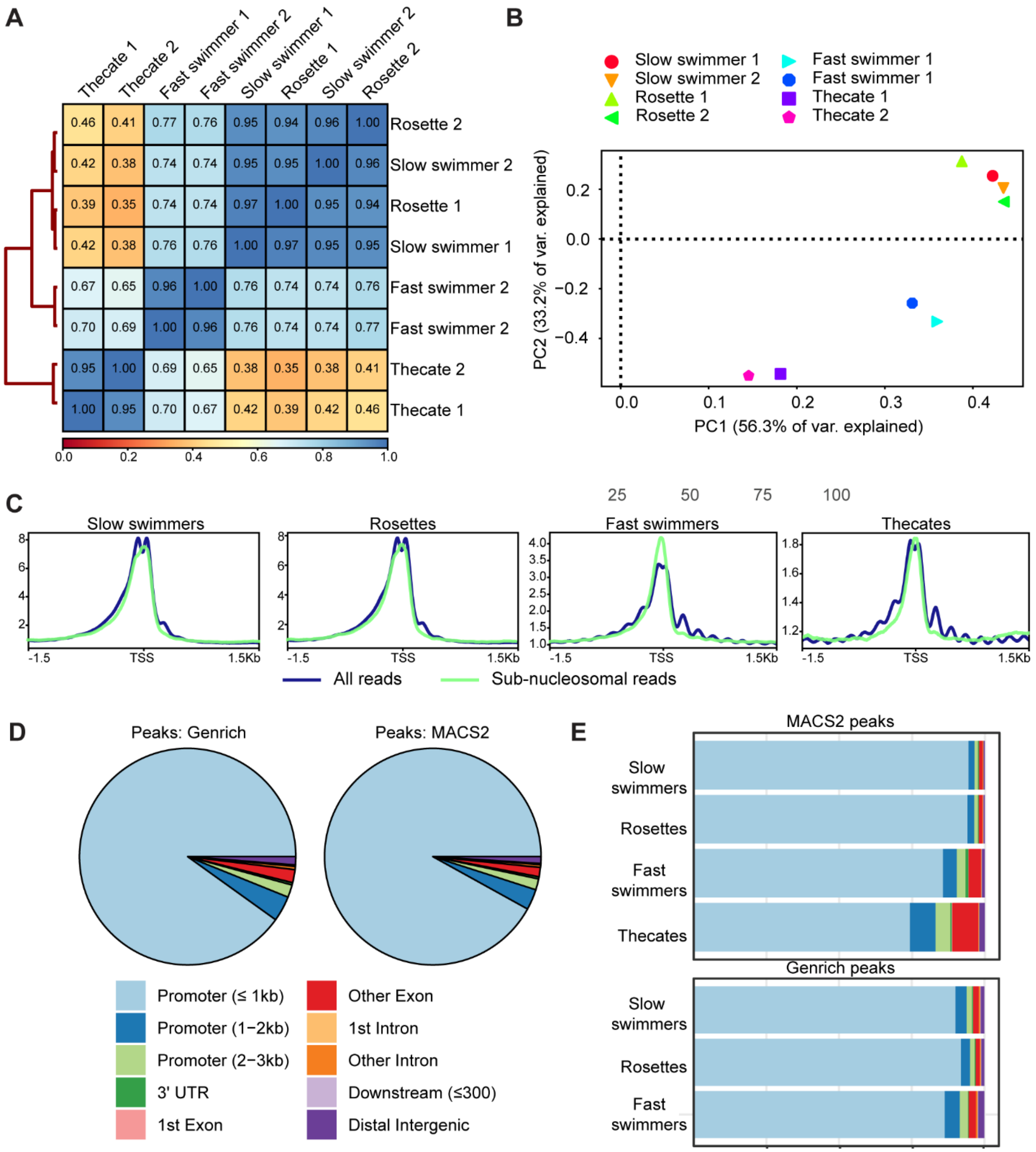

Figure S1. Additional ATAC-seq analysis. (**A**) Clustered heatmap showing the correlation coefficients between samples based on the Pearson method. (**B**) PCA plot on all samples showing the two first principal components. (**C**) Metaplot of ATAC-seq read density around the TSS's of all genes comparing all reads to sub-nucleosome sized fragments. **(D)** ChIPseeker annotation of peaks from either Genrich or MACS2. For MACS2 peaks from all cell types are shown while for Genrich all cell types except thecate cells were used (**E**) ChIPseeker annotation of peaks showing each cell type separately.

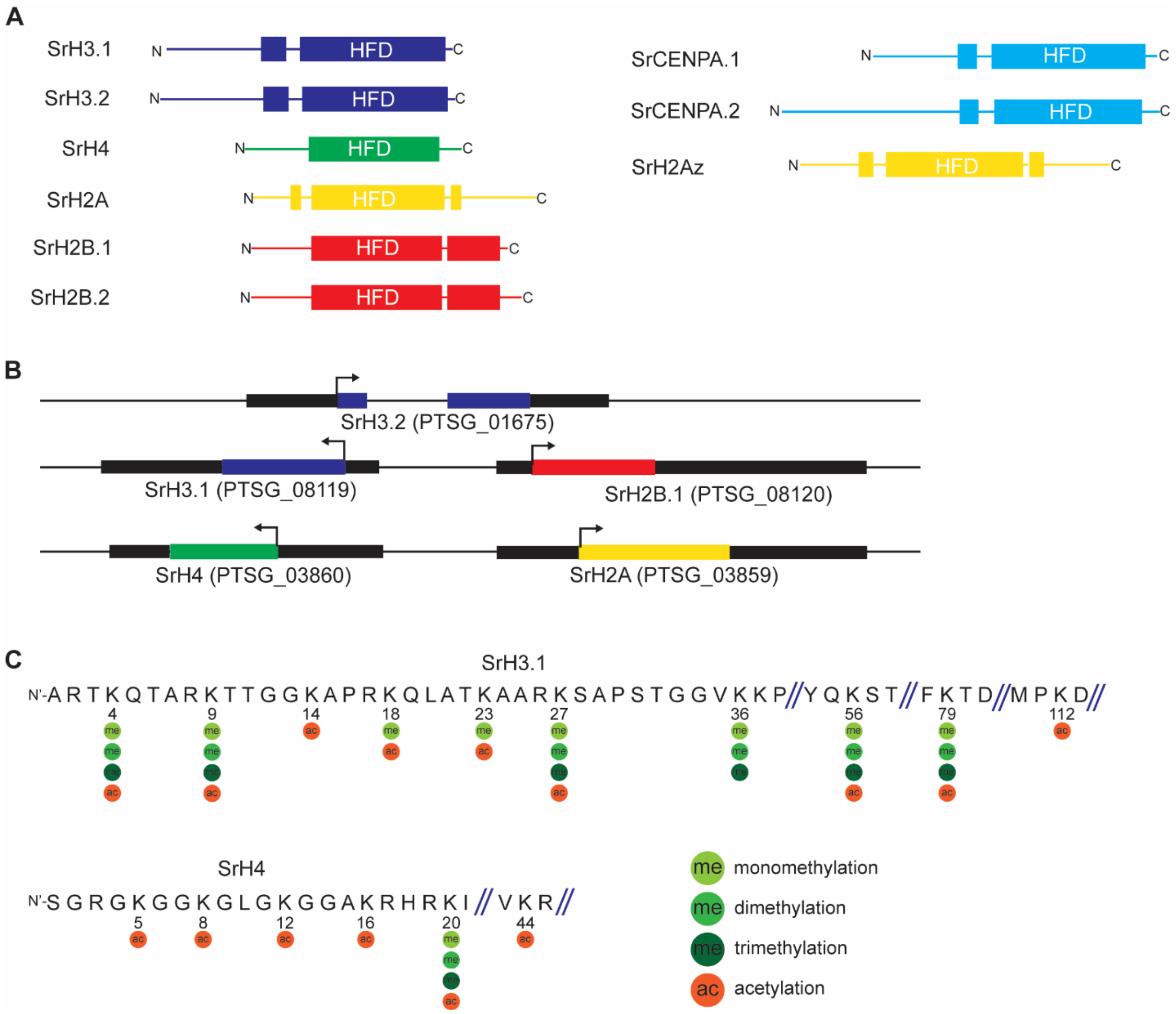

Figure S2. The histone PTM complement of *S. rosetta*. (A) Schematic models of all histone proteins annotated in *S. rosetta*. We annotated the histone complement using blast searches as well as previously published phylogenetic analyses (Grau-Bové et al. 2021). S. rosetta has two H3 variants which are putatively annotated as a replication-dependent SrH3.1 and a replication-independent SrH3.2. This is due to SrH3.1 being in a cluster with SrH2A as expected for a canonical replication-dependent histone while SrH3.2 is a single gene which also contains an intron characteristic of a replication-independent variant (see **B**) (Henikoff and Smith 2015). S. rosetta has a single H4 protein, two H2B variants with a variable c-terminal length and a single H2A which is a H2Ax based on the presence of characteristic SQEY amino-acid motif in the C-terminal region. In addition to the canonical histones, *S. rosetta* possesses two centromeric histone H3 variants, SrCENPA.1 and SrCENPA.2 and an H2Az homolog, SrH2Az. (**B**) Genomic organization of the single *SrH3.2* gene and examples of *SrH3.1*, *SrH4*, *SrH2B.1* and *SrH2A*. *Sr.H3.2* is a single copy in the genome and contains an intron. SrH3.1 and SrH2B are in a cluster as are Sr.H4 and SrH2A. (**C**) Schematic of part of the HrH3.1 and SrH4 protein sequence showing positions where lysine methylation and acetylation were identified by mass-spectrometry.

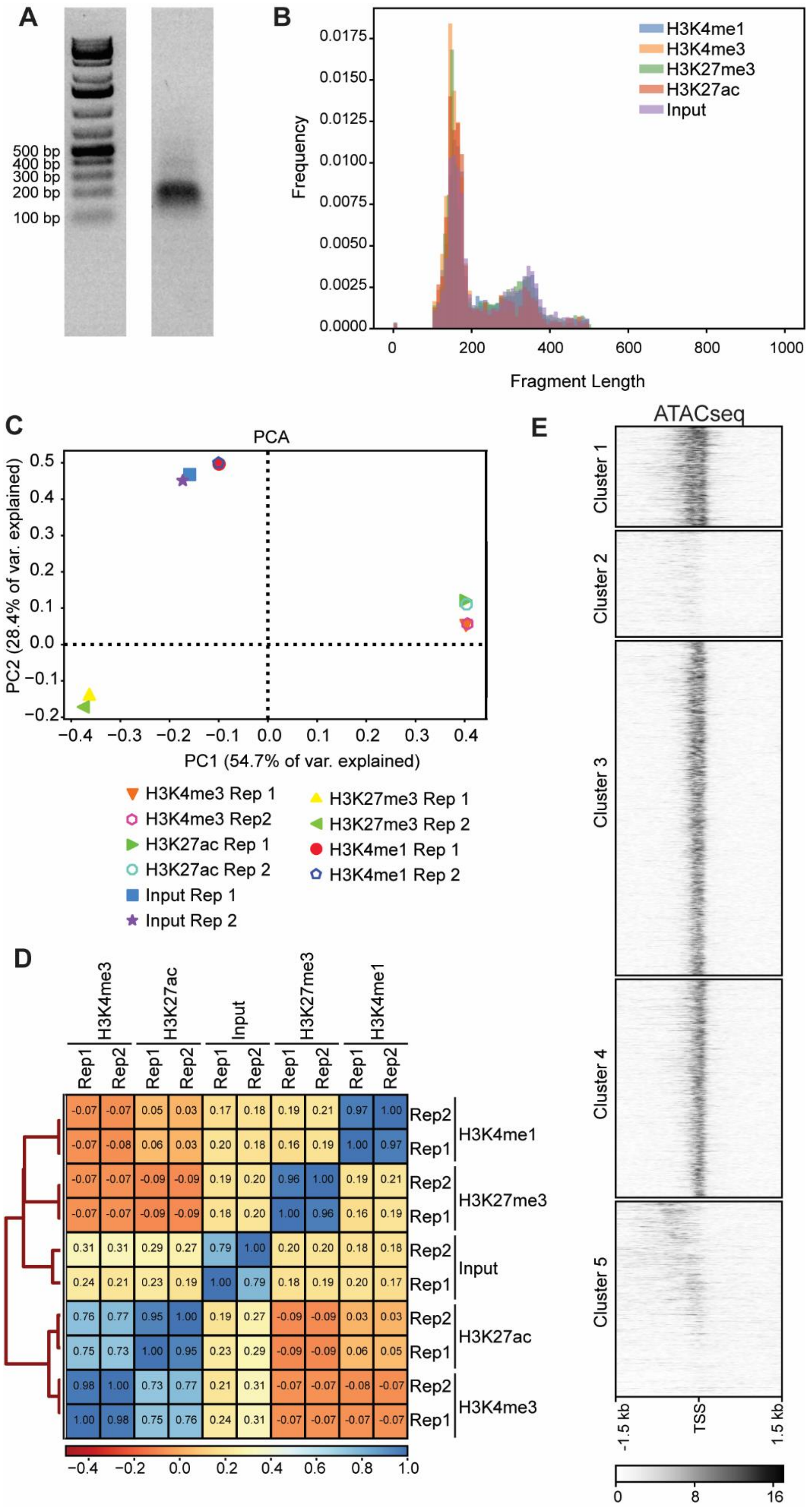
A
500 bp
400 bp
300 bp
200 bp
100 bp
B
H3K4me1
H3K4me3
H3K27me3
H3K27ac
Input
Frequency
Fragment Length
C
PCA
PC2 (28.4% of var. explained)
PC1 (54.7% of var. explained)
H3K4me3 Rep 1
H3K4me3 Rep2
H3K27ac Rep 1
H3K27ac Rep 2
Input Rep 1
Input Rep 2
H3K27me3 Rep 1
H3K27me3 Rep 2
H3K4me1 Rep 1
H3K4me1 Rep 2
D
H3K4me3
H3K27ac
Input
H3K27me3
H3K4me1
Rep1
Rep2
-0.07 -0.07 0.05 0.03 0.17 0.18 0.19 0.21 0.97 1.00 Rep2 H3K4me1
-0.07 -0.08 0.06 0.03 0.20 0.18 0.16 0.19 1.00 0.97 Rep1
-0.07 -0.07 -0.09 -0.09 0.19 0.20 0.96 1.00 0.19 0.21 Rep2 H3K27me3
-0.07 -0.07 -0.09 -0.09 0.18 0.20 1.00 0.96 0.16 0.19 Rep1
0.31 0.31 0.29 0.27 0.79 1.00 0.20 0.20 0.18 0.18 Rep2 Input
0.24 0.21 0.23 0.19 1.00 0.79 0.18 0.19 0.20 0.17 Rep1
0.76 0.77 0.95 1.00 0.19 0.27 -0.09 -0.09 0.03 0.03 Rep2 H3K27ac
0.75 0.73 1.00 0.95 0.23 0.29 -0.09 -0.09 0.06 0.05 Rep1
0.98 1.00 0.73 0.77 0.21 0.31 -0.07 -0.07 -0.08 -0.07 Rep2 H3K4me3
1.00 0.98 0.75 0.76 0.24 0.31 -0.07 -0.07 -0.07 -0.07 Rep1
−0.4 −0.2 0.0 0.2 0.4 0.6 0.8 1.0
E
ATACseq
Cluster 1
Cluster 2
Cluster 3
Cluster 4
Cluster 5
-1.5 kb
TSS
1.5 kb
0
8
16

Figure S3. Additional ChIP-seq data. (**A**) Gel electrophoresis of DNA from a nucleosome extraction used for ChIP-seq. The majority of fragments are approximately mono-nucleosome size (~140bps). (**B**) Frequency plot showing the size of sequenced fragments from ChIP-seq data. A bin size of 10bp was used. In all 4 antibody ChIPs and input the majority of fragments are below 200bps, as expected for mono-nucleosome fragments. (**C**) PCA plot on all ChIP-seq samples showing the high correlation between replicates. (**D**) Clustered heatmap showing the correlation coefficients between samples based on the Pearson method. (**E**) Heatmap showing ATAC-seq in Slow Swimmers for all genes divided by cluster. Genes are ordered as in Figure 4B. Cluster 1 (n= 1232), Cluster 2 (n= 1305), Cluster 3 (n= 4112), Cluster 4 (n= 2681), Cluster 5 (n= 2401),

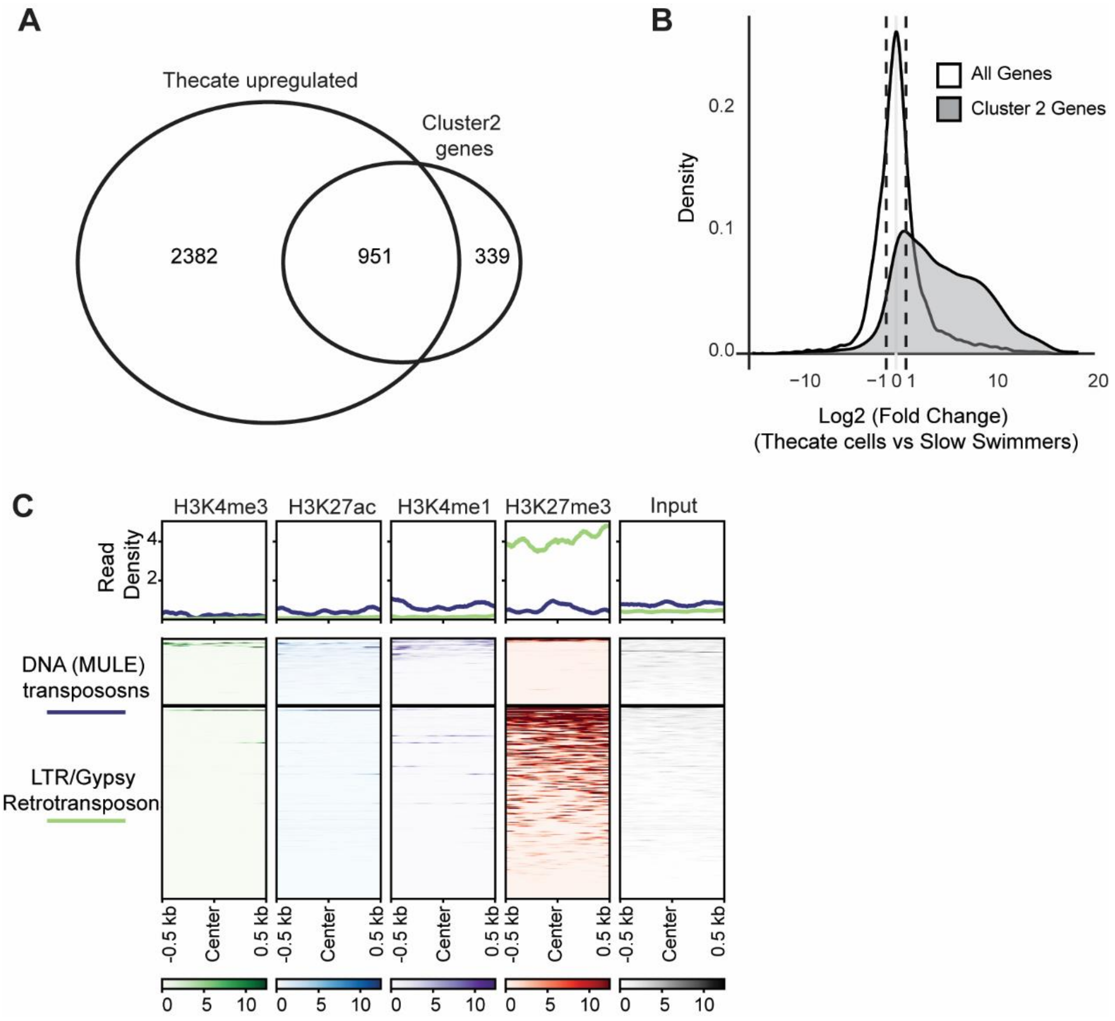

Figure S4. Additional analysis of H3K27me3 marked regions. (**A**) Venn diagram showing the overlap between genes from Cluster 2 and genes upregulated in thecate cells vs slow swimmers. Log2 fold change of +/- 1, as determined using DEseq2, was considered as a significant change. (**B**) Density plot showing the log2 fold change between thecate cells and slow swimmers for all genes and for the cluster 2 genes. Log_2 fold change was calculated using DESeq2. (**C**) Metaplot and heatmap showing ChIP-seq for the indicated antibodies and input over DNA(MULE) transposons (n=94) and LTR/Gypsy transposons (n=269). Reads here were the same as used in all other analyses (i.e. including base quality cut-off).

Supplementary Table 1. Histone genes present in *S. rosetta* and their genomic locations.

Histone (protein)	Gene names	Genomic location (Supercontig:position)
SrH3.1	PTSG_08119	GL832975:510871-511825
	PTSG_10057	GL832986:418356-419309
Sr.H3.2	PTSG_01675	GL832957:484880-486601
SrH4	PTSG_03860	GL832962:1811404-1812225
	PTSG_00674	GL832956:439532-440249
SrH2A	PTSG_03859	GL832962:1810216-1811282
	PTSG_00675	GL832956:440469-441465
SrH2Az	PTSG_11229	GL832996:49505-51660
SrH2B	PTSG_08120	GL832975:512187-513213
	PTSG_10056	GL832986:416738-417364
SrCENPA.1	PTSG_09298	GL832982:51979-53872
SrCENPA.2	PTSG_08448	GL832977:831645-832995

Supplementary Table 2. List of antibodies used in this study.

Target	Source	Concentration used
H3K27me3	(Rose et al. 2016)	5ul per ChIP
H3K4me3	(Farcas et al. 2012)	5ul per ChIP
H3K4me1	Cell Signalling Technologies D1A9	5ul per ChIP
H3K27ac	Cell Signalling Technologies D5E4	5ul per ChIP

Supplementary Table 3. List of software used.

Software	Reference	Website
Illumina BCL Convert v4		https://support-docs.illumina.com/SW/BCL_Convert_v4.0/Content/SW/BCLConvert/BCLConvert.htm
Bedtools v2.26.0	(Quinlan and Hall 2010)	https://bedtools.readthedocs.io/en/latest/
Trimmomatic v0.39	(Bolger et al. 2014)	http://www.usadellab.org/cms/?page=trimmomatic
Bowtie2 v2.3.4.1	(Langmead and Salzberg 2012)	http://bowtie-bio.sourceforge.net/bowtie2/index.shtml
Picard v2.18.26		https://broadinstitute.github.io/picard/
SAMtools v1.7	(Li et al. 2009)	https://www.htslib.org/
deepTools v3.4.3	(Ramírez et al. 2014)	https://deeptools.readthedocs.io/en/develop/
Macs2 v2.1.1	(Zhang et al. 2008)	https://pypi.org/project/MACS2/
Genrich v0.6.2		https://github.com/jsh58/Genrich

STAR v020101	(Dobin et al. 2013)	https://github.com/alexdobin/STAR
ChIPseeker	(Yu et al. 2015)	https://www.bioconductor.org/packages/release/bioc/html/ChIPseeker.html
DESeq2		https://bioconductor.org/packages/release/bioc/html/DESeq2.html
GenomicRanges	(Lawrence et al. 2013)	https://bioconductor.org/packages/release/bioc/html/GenomicRanges.html
RepeatMasker	(Smit 2013-2015)	https://www.repeatmasker.org/
IGV	(Robinson et al. 2011)	https://igv.org/
ggplot2	(Wickham 2016)	https://ggplot2.tidyverse.org/
EpiProfile2.0	(Yuan et al. 2018)	https://github.com/zfyuan/EpiProfile2.0_Family

Supplementary references

Bolger AM, Lohse M, Usadel B. 2014. Trimmomatic: a flexible trimmer for Illumina sequence data. *Bioinformatics* **30**: 2114-2120.

Dobin A, Davis CA, Schlesinger F, Drenkow J, Zaleski C, Jha S, Batut P, Chaisson M, Gingeras TR. 2013. STAR: ultrafast universal RNA-seq aligner. *Bioinformatics* **29**: 15-21.

Farcas AM, Blackledge NP, Sudbery I, Long HK, McGouran JF, Rose NR, Lee S, Sims D, Cerase A, Sheahan TW et al. 2012. KDM2B links the Polycomb Repressive Complex 1 (PRC1) to recognition of CpG islands. *Elife* **1**: e00205.

Grau-Bové X, Navarrete C, Chiva C, Pribasnig T, Antó M, Torruella G, Galindo LJ, Lang BF, Moreira D, López-Garcia P et al. 2021. Comparative proteogenomics deciphers the origin and evolution of eukaryotic chromatin. *bioRxiv*: 2021.2011.2030.470311.

Henikoff S, Smith MM. 2015. Histone variants and epigenetics. *Cold Spring Harb Perspect Biol* **7**: a019364.

Langmead B, Salzberg SL. 2012. Fast gapped-read alignment with Bowtie 2. *Nature methods* **9**: 357-359.

Lawrence M, Huber W, Pages H, Aboyoun P, Carlson M, Gentleman R, Morgan MT, Carey VJ. 2013. Software for computing and annotating genomic ranges. *PLoS Comput Biol* **9**: e1003118.

Li H, Handsaker B, Wysoker A, Fennell T, Ruan J, Homer N, Marth G, Abecasis G, Durbin R, Subgroup GPDP. 2009. The sequence alignment/map format and SAMtools. *bioinformatics* **25**: 2078-2079.

Quinlan AR, Hall IM. 2010. BEDTools: a flexible suite of utilities for comparing genomic features. *Bioinformatics* **26**: 841-842.

Ramírez F, Dündar F, Diehl S, Grüning BA, Manke T. 2014. deepTools: a flexible platform for exploring deep-sequencing data. *Nucleic acids research* **42**: W187-W191.

Robinson JT, Thorvaldsdóttir H, Winckler W, Guttman M, Lander ES, Getz G, Mesirov JP. 2011. Integrative genomics viewer. *Nature biotechnology* **29**: 24-26.

Rose NR, King HW, Blackledge NP, Fursova NA, Ember KJ, Fischer R, Kessler BM, Klose RJ. 2016. RYBP stimulates PRC1 to shape chromatin-based communication between Polycomb repressive complexes. *Elife* **5**.

Smit A, Hubley, R & Green, P. 2013-2015.

Wickham H. 2016. ggplot2: Elegant Graphics for Data Analysis. Springer-Verlag, New York. ISBN 978-3-319-24277-4.

Yu G, Wang L-G, He Q-Y. 2015. ChIPseeker: an R/Bioconductor package for ChIP peak annotation, comparison and visualization. *Bioinformatics* **31**: 2382-2383.

Yuan ZF, Sidoli S, Marchione DM, Simithy J, Janssen KA, Szurgot MR, Garcia BA. 2018. EpiProfile 2.0: A Computational Platform for Processing Epi-Proteomics Mass Spectrometry Data. *J Proteome Res* **17**: 2533-2541.

Zhang Y, Liu T, Meyer CA, Eeckhoute J, Johnson DS, Bernstein BE, Nusbaum C, Myers RM, Brown M, Li W. 2008. Model-based analysis of ChIP-Seq (MACS). *Genome biology* **9**: 1-9.
